# FOXO3a-mediated suppression of the self-renewal capacity of sphere-forming cells derived from the ovarian cancer SKOV3 cell line by 7-difluoromethoxyl-5,4’-di-n-octyl genistein

**DOI:** 10.3892/mmr.2021.12449

**Published:** 2021-09-17

**Authors:** Yingxia Ning, Chaoyuan Luo, Kaiqun Ren, Meifang Quan, Jianguo Cao

Mol Med Rep 9: 1982-1988, 2014; DOI: 10.3892/mmr.2014.2012

Subsequently to the publication of this paper, an interested reader drew to the authors’ attention that strikingly similar western blot data were shown in Fig. 2 (to portray the Nagon data in Fig. 2A and the CD133 data in Fig. 2B), and the same data also appeared to have been included in Fig. 4 (to show the p-FOXO3a data). After having examined their original data, the authors have realized that these figures were inadvertently assembled incorrectly

The corrected versions of Figs. 2 and 4, showing the correct data for the CD133 experiment in Fig. 2B and the p-FOXO3a experiment in Fig. 4, are shown opposite. Note that these errors did not significantly affect the results or the conclusions reported in this paper, and all the authors agree to this Corrigendum. Furthermore, the authors apologize to the readership for any inconvenience caused.

## Figures and Tables

**Figure 2.**
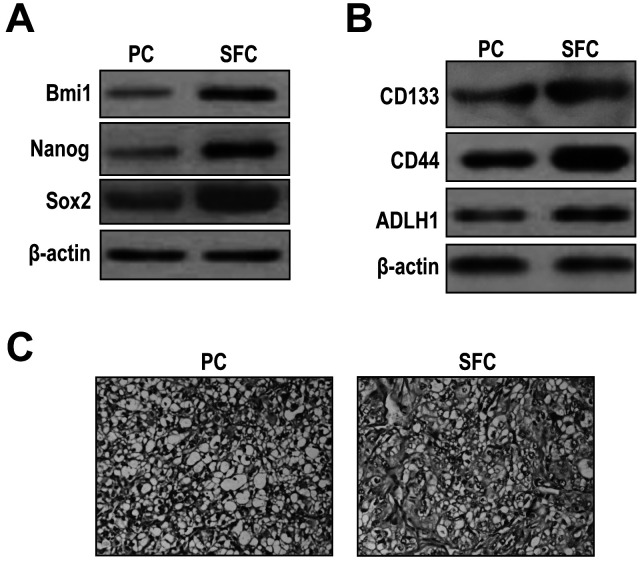
SKOV3 cell-line-derived SFCs possess properties of OCSLCs. (A) Western immunoblot analysis demonstrated that BMI1, Nagon and SOX2 were highly expressed in SFCs derived from SKOV3 cells compared with the PC. (B) Western immunoblot analysis demonstrated that CD133, CD44 and ADLH1 were highly expressed in SFCs derived from SKOV3 cells compared with the PC. (C) H&E staining revealed that the histological features of SFC-derived xenografted tumors were similar to those identified for parental SKOV3 cells (magnification, ×100). SFCs, sphere-forming cells; OCSLCs, ovarian cancer stem-like cells; PC, parental cells.

**Figure 4.**
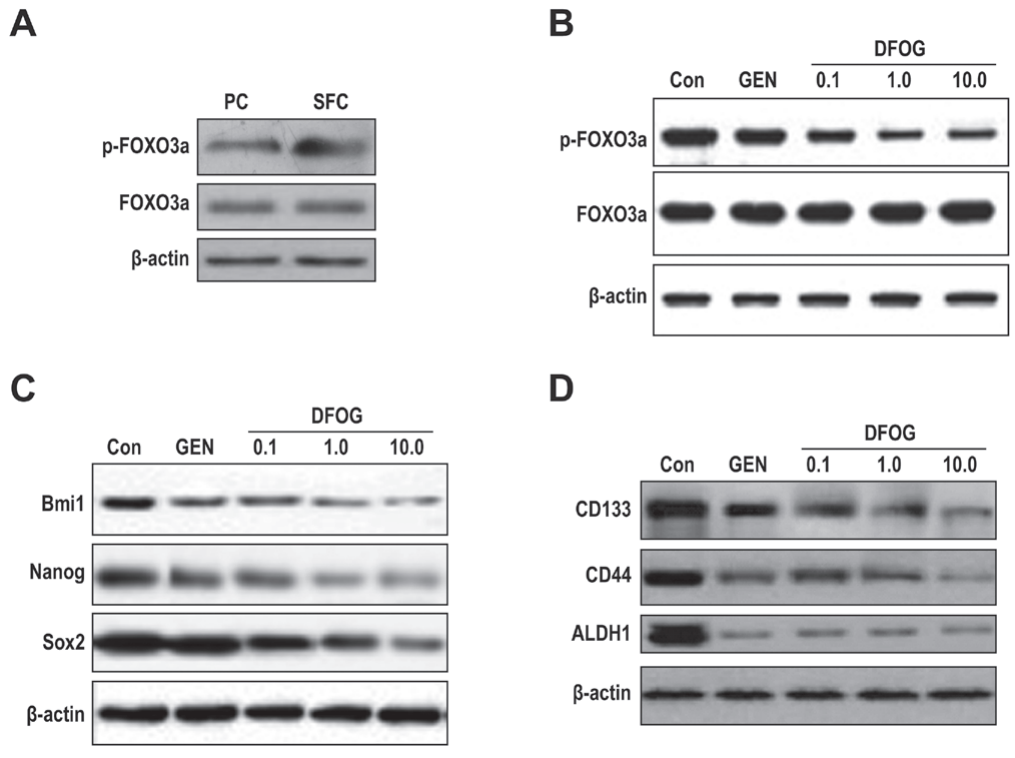
DFOG reduces FOXO3a phosphorylation and CSC marker expression of OCSLCs derived from SKOV3 cells. (A) The phosphorylated form of the FOXO3a protein was highly expressed in SFCs derived from SKOV3 cells compared with corresponding PCs. (B) Treatment with DFOG downregulated the expression of phosphorylated FOXO3a in SFCs derived from SKOV3 cells. (C) Treatment with DFOG downregulated the expression of the self-renewal associated proteins, including BMI1, Nagon and SOX2 in SFCs derived from SKOV3 cells. (D) Treatment with DFOG downregulated the expression of CSC markers, including CD133, CD44 and ALDH1 in SFCs derived from SKOV3 cells. DFOG, 7-difluoromethoxyl-5,4’-di-n-octyl genistein; CSC, cancer stem cell; OCSLCs, ovarian cancer stem-like cells; SFCs, sphere-forming cells; PCs, parental cells.

